# 2-Amino-5-nitro­phenyl 2-chloro­phenyl ketone

**DOI:** 10.1107/S160053680902755X

**Published:** 2009-07-18

**Authors:** Jerry P. Jasinski, Ray J. Butcher, Q. N. M. Hakim Al-Arique, H. S. Yathirajan, A. R. Ramesha

**Affiliations:** a Department of Chemistry, Keene State College, 229 Main Street, Keene, NH 03435-2001, USA; b Department of Chemistry, Howard University, 525 College Street NW, Washington DC 20059, USA; c Department of Studies in Chemistry, University of Mysore, Manasagangotri, Mysore 570 006, India; d RL Fine Chem, Bangalore 560 064, India

## Abstract

In the title compound, C_13_H_9_ClN_2_O_3_, an intra­molecular hydrogen bond between the carbonyl O and an amine H atom from the 2-amino­benzoyl group stabilizes the mol­ecule, keeping these two groups nearly in the same plane [dihedral angle 14.6 (6)°]. The dihedral angle between the mean planes of the planar 2-amino­benzoyl and 2-chloro­benzoyl groups is 73.8 (6)°. The crystal packing is stabilized by a collection of inter­mediate hydrogen-bonding inter­actions which forms an infinite N—H⋯O⋯H—N—H⋯O hydrogen-bonded chain along the *c* axis in concert with weak N—H⋯Cl inter­actions in the same direction, producing a two-dimensional inter­molecular bonding network parallel to (001). Additional weak C—Cl⋯*Cg* [Cl⋯*Cg* = 3.858 (3) Å] and N—O⋯*Cg* [O⋯*Cg* = 3.574 (1) and 3.868 (6) Å] π-ring inter­actions provide added support to the crystal stability. A MOPAC computational calculation gives support to these observations.

## Related literature

For related structures, see: Cox *et al.* (1997 ▶, 2008 ▶); Harrison *et al.* (2005 ▶); Malathy Sony *et al.* (2005 ▶); Prasanna & Guru Row (2000 ▶); Xing *et al.* (2005 ▶). For background to benzophenone derivatives, see: Colpaert *et al.* (2004 ▶); Deleu *et al.* (1992 ▶); Duncan *et al.* (2004 ▶); Evans *et al.* (1987 ▶); Ottosen *et al.* (2003 ▶); Revesz *et al.* (2004 ▶); Sieroń *et al.* (2004 ▶); Wiesner *et al.* (2002 ▶). For a description of the Cambridge Structural Database, see: Allen (2002 ▶). For MOPAC AM1 computational calculations, see: Schmidt & Polik (2007 ▶).
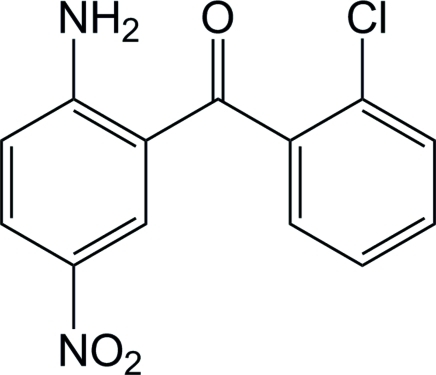


## Experimental

### 

#### Crystal data

C_13_H_9_ClN_2_O_3_
*M* *_r_* = 276.67
Monoclinic, 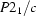

*a* = 10.6120 (3) Å
*b* = 11.3314 (3) Å
*c* = 10.8456 (3) Å
β = 108.399 (3)°
*V* = 1237.50 (6) Å^3^
*Z* = 4
Mo *K*α radiation
μ = 0.31 mm^−1^
*T* = 110 K
0.47 × 0.36 × 0.28 mm

#### Data collection

Oxford Diffraction Gemini R CCD diffractometer
Absorption correction: multi-scan (*CrysAlis RED*; Oxford Diffraction, 2007 ▶) *T*_min_ = 0.868, *T*_max_ = 0.916
8758 measured reflections
4131 independent reflections
3069 reflections with *I* > 2σ(*I*)
*R*_int_ = 0.021

#### Refinement

*R*[*F* ^2^ > 2σ(*F* ^2^)] = 0.036
*wR*(*F* ^2^) = 0.099
*S* = 1.04
4131 reflections
172 parameters
H-atom parameters constrained
Δρ_max_ = 0.42 e Å^−3^
Δρ_min_ = −0.27 e Å^−3^

### 

Data collection: *CrysAlisPro* (Oxford Diffraction, 2007 ▶); cell refinement: *CrysAlisPro*; data reduction: *CrysAlisPro*; program(s) used to solve structure: *SHELXS97* (Sheldrick, 2008 ▶); program(s) used to refine structure: *SHELXL97* (Sheldrick, 2008 ▶); molecular graphics: *SHELXTL* (Sheldrick, 2008 ▶); software used to prepare material for publication: *SHELXTL*.

## Supplementary Material

Crystal structure: contains datablocks global, I. DOI: 10.1107/S160053680902755X/kj2123sup1.cif

Structure factors: contains datablocks I. DOI: 10.1107/S160053680902755X/kj2123Isup2.hkl

Additional supplementary materials:  crystallographic information; 3D view; checkCIF report

## Figures and Tables

**Table 1:** Hydrogen-bond geometry (Å, °)

*D*—H⋯*A*	*D*—H	H⋯*A*	*D*⋯*A*	*D*—H⋯*A*
N1—H1*A*⋯O3^i^	0.88	2.22	3.0733 (12)	164
N1—H1*B*⋯O3	0.88	2.07	2.7176 (12)	130
N1—H1*B*⋯Cl^ii^	0.88	2.71	3.4848 (10)	148
C13—H13*A*⋯O2^iii^	0.95	2.46	3.1862 (14)	133

Symmetry codes: (i) 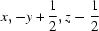; (ii) 
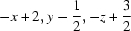
; (iii) 
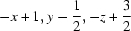
.

**Fig. 1.**
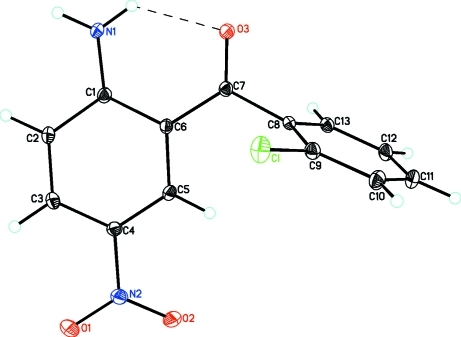
Molecular structure of the title compound showing the atom labeling scheme and 50% probability displacement ellipsoids. Dashed lines indicate hydrogen bonds.

**Fig. 2.**
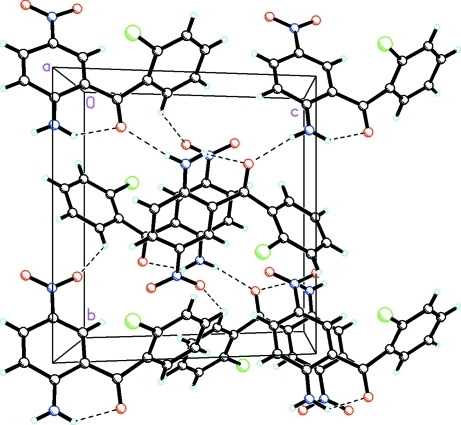
Packing diagram of the title compound, viewed down the a axis. Dashed lines indicate intramolecular N-H···O and intermediate intermolecular N-H···O and C-H···O hydrogen bonds in concert with weak N-H···Cl interactions in the same direction producing an infinite, 2-dimensional intermolecular bonding network parallel to the (001) plane of the unit cell.

## supplementary crystallographic information

### Comment

Benzophenone derivatives are widely used in sunscreen lotions for UVA protection (Deleu *et al.*, 1992).  Benzophenone and related analogues have been reported to act as antiallergic, anti-inflammatory, antiasthamatic, antimalarial, anti-microbial and antianaphylactic agents (Evans *et al.*, 1987; Wiesner *et al.*, 2002; Sieroń *et al.*, 2004). The competence of benzophenones as chemotherapeutic agents, especially as inhibitors of HIV-1 reverse transcriptase RT, cancer and inflammation, is well established and their chemistry has been studied extensively (Revesz *et al.*, 2004). Phenylmethanones are a class of compounds having many pharmacological properties. 4-Aminobenzophenones have high anti-inflammatory activity (Ottosen *et al.*, 2003), a benzophenyl cyano derivative acts as a vasorelaxant (Duncan *et al.*, 2004) and the piperidinyl derivative produces analgesia (Colpaert *et al.*, 2004). The crystal structures of  some related compounds, viz., N-(2-benzoyl-4-chlorophenyl)-2-chloroacetamide (Malathy Sony *et al.*, 2005), 2-chloroacetamido-5-chlorobenzophenone and 2-chloroacetamido-5-chloro-2'-fluorobenzophenone (Prasanna & Guru Row, 2000), 2-methylamino-5-chlorobenzophenone (Cox *et al.*, 1997), 2-amino-2'-chloro-5-methylbenzophenone (Xing *et al.*, 2005) and 3-chloro-4-hydroxy-4'-methylbenzophenone (Harrison *et al.*, 2005) have been reported. Over 450 crystal structures of benzophenone derivatives in the Cambridge Structural Database (CSD, Version 5.26; Allen, 2002) highlight the importance of structural studies on such pharmaceutically useful compounds. The title compound, C_13_H_9_ClN_2_O_3_, is an intermediate in the synthesis of certain anxiolytic, anticonvulsant and sedative drugs. The title compound is also a starting material for the synthesis of diazepam and other benzodiazepines. In view of the importance of the title compound, the present paper describes its crystal structure.

The title compound, C_13_H_9_ClN_2_O_3_, crystallizes with one molecule in the asymmetric unit with Z = 4. An intramolecular hydrogen bond between the carbonyl oxygen (O3) and an amine hydrogen atom (H1B) from the 2-amino-5-nitrobenzoyl group keeps these two groups nearly in the same plane releative to each other (Fig. 1). The dihedral angle between the mean planes of the carbony group (-C6-C7(O3)-C8-) and the mean planes of the 2-amino and 2'-chlorobenzoyl planar groups is 14.6 (6)° and 66.2 (9)°, respectively. The C5-C6-C7-O3 torsion angle (164.45 (11)°) supports this observation. The nitro group is twisted slightly away from the plane of the 2-amino-benzyl group (O2-N2-C4-C3 torsion angle = 178.46 (11)°).  The dihedral angle between the mean planes of the 2-aminobenzyl and 2'-chlorobenzyl planar groups is 73.8 (6)°.  This value lies between the large twist angle of 83.72 (6)° as seen in 2-amino and 2'-chlorobenzenophenone, C_13_H_9_Cl_2_NO, (Cox *et al.*, 2008) and 64.66 (8)° observed in 4-chloro-4'-hydroxybenzophenone, C_13_H_9_ClO_2_ (Cox *et al.*, 2008).  Crystal packing is supported by a collection of intermediate N1-H1A···O3, N1-H1B···O3, C13-H13A···O2 hydrogen bonds and weak N1-H1B···Cl intermolecular interactions (see Table 1) which produces an infinite, two-dimensional N-H···O···H-N-H···O bonding network parallel to the (001) plane of the unit cell (Fig. 2). Additional weak C9-Cl···Cg(2) [Cl···Cg(2)= 3.858 (3)Å], N2-O1···Cg(2) [O1···Cg(2) = 3.574 (1)Å] and N2-O2···Cg(1) [O2···Cg(1) = 3.868 (6)Å] π-ring interactions (-x, 1-y, -x; x, 1/2-y, 1/2+z; 1-x, 1-y, 1-z; Cg(1) = C1-C6 and Cg(2) = C8-C13 centroids, respectively) collectively provide added support to crystal stability.

After a MOPAC AM1 computational calculation (Schmidt & Polik, 2007), the nitro group now lies in the plane of the 2-aminobenzoyl group.  The dihedral angle between the mean planes of the 2-aminobenzoyl and 2'-chlorobenzoyl planar groups becomes 88.6 (1)° while the dihedral angle between the mean planes of the carbony group (-C6-C7(O3)-C8-) and the mean planes of the 2-amino and 2'-chlorobenzoyl planar groups becomes 19.7 (3)° and 81.7 (1)°, respectively. This supports the observation of a collective action of intermediate N-H···O, C-H···O hydrogen bonds, weak N-H···Cl intermolecular interactions and weak C-Cl···Cg, N-O···Cg π-ring interactions influencing crystal packing stability.

### Experimental

The title compound was obtained as a gift sample from *R. L*. Fine Chem, Bangalore, India. The compound was used without further purification. Pale yellow crystals (m.p. 378–380 K) were obtained by slow evaporation from acetonitrile solution.

### Refinement

All of the H atoms were placed in their calculated positions and then refined using the riding model with N—H = 0.88, C—H = 0.95 Å, and with *U*_iso_(H) = 1.18–1.21*U*_eq_(C,*N*).

### Crystal data

**Table:**

C_13_H_9_ClN_2_O_3_	*F*(000) = 568
*M**_r_* = 276.67	*D*_x_ = 1.485 Mg m^−^^3^
Monoclinic, *P*2_1_/*c*	Mo *K*α radiation, λ = 0.71073 Å
Hall symbol: -P 2ybc	Cell parameters from 4505 reflections
*a* = 10.6120 (3) Å	θ = 4.7–32.6°
*b* = 11.3314 (3) Å	µ = 0.31 mm^−^^1^
*c* = 10.8456 (3) Å	*T* = 110 K
β = 108.399 (3)°	Chunk, colorless
*V* = 1237.50 (6) Å^3^	0.47 × 0.36 × 0.28 mm
*Z* = 4	

### Data collection

**Table:**

Oxford Diffraction Gemini R CCD diffractometer	4131 independent reflections
Radiation source: fine-focus sealed tube	3069 reflections with *I* > 2σ(*I*)
graphite	*R*_int_ = 0.021
Detector resolution: 10.5081 pixels mm^-1^	θ_max_ = 32.7°, θ_min_ = 4.7°
φ and ω scans	*h* = −15→16
Absorption correction: multi-scan (*CrysAlis RED*; Oxford Diffraction, 2007)	*k* = −13→16
*T*_min_ = 0.868, *T*_max_ = 0.916	*l* = −16→11
8758 measured reflections	

### Refinement

**Table:**

Refinement on *F*^2^	Primary atom site location: structure-invariant direct methods
Least-squares matrix: full	Secondary atom site location: difference Fourier map
*R*[*F*^2^ > 2σ(*F*^2^)] = 0.036	Hydrogen site location: inferred from neighbouring sites
*wR*(*F*^2^) = 0.099	H-atom parameters constrained
*S* = 1.04	*w* = 1/[σ^2^(*F*_o_^2^) + (0.0548*P*)^2^] where *P* = (*F*_o_^2^ + 2*F*_c_^2^)/3
4131 reflections	(Δ/σ)_max_ < 0.001
172 parameters	Δρ_max_ = 0.42 e Å^−^^3^
0 restraints	Δρ_min_ = −0.27 e Å^−^^3^

### Special details

**Table:**

Geometry. All e.s.d.'s (except the e.s.d. in the dihedral angle between two l.s. planes) are estimated using the full covariance matrix. The cell e.s.d.'s are taken into account individually in the estimation of e.s.d.'s in distances, angles and torsion angles; correlations between e.s.d.'s in cell parameters are only used when they are defined by crystal symmetry. An approximate (isotropic) treatment of cell e.s.d.'s is used for estimating e.s.d.'s involving l.s. planes.
Refinement. Refinement of *F*^2^ against ALL reflections. The weighted *R*-factor *wR* and goodness of fit *S* are based on *F*^2^, conventional *R*-factors *R* are based on *F*, with *F* set to zero for negative *F*^2^. The threshold expression of *F*^2^ > σ(*F*^2^) is used only for calculating *R*-factors(gt) *etc*. and is not relevant to the choice of reflections for refinement. *R*-factors based on *F*^2^ are statistically about twice as large as those based on *F*, and *R*- factors based on ALL data will be even larger.

### Fractional atomic coordinates and isotropic or equivalent isotropic displacement parameters (Å^2^)

**Table:**

	*x*	*y*	*z*	*U*_iso_*/*U*_eq_	
Cl	0.96431 (3)	0.63182 (3)	0.78827 (3)	0.02769 (10)	
O1	0.48712 (10)	0.77733 (9)	0.32532 (9)	0.0350 (2)	
O2	0.50201 (9)	0.76958 (8)	0.52898 (8)	0.0271 (2)	
O3	0.82129 (9)	0.31290 (8)	0.73196 (7)	0.02303 (19)	
N1	0.82197 (10)	0.31577 (9)	0.48161 (9)	0.0214 (2)	
H1A	0.8367	0.2872	0.4120	0.026*	
H1B	0.8515	0.2779	0.5561	0.026*	
N2	0.52756 (10)	0.73094 (9)	0.43331 (9)	0.0211 (2)	
C1	0.75466 (11)	0.41681 (10)	0.47433 (10)	0.0162 (2)	
C2	0.70760 (12)	0.47688 (11)	0.35352 (10)	0.0197 (2)	
H2A	0.7267	0.4453	0.2802	0.024*	
C3	0.63591 (12)	0.57843 (11)	0.33976 (10)	0.0202 (2)	
H3A	0.6049	0.6169	0.2579	0.024*	
C4	0.60840 (11)	0.62550 (10)	0.44837 (10)	0.0169 (2)	
C5	0.65568 (10)	0.57301 (10)	0.56856 (10)	0.0157 (2)	
H5A	0.6376	0.6076	0.6410	0.019*	
C6	0.73013 (10)	0.46916 (10)	0.58484 (10)	0.0148 (2)	
C7	0.77971 (11)	0.41506 (10)	0.71401 (10)	0.0159 (2)	
C8	0.77845 (11)	0.48654 (10)	0.83067 (9)	0.0154 (2)	
C9	0.85984 (11)	0.58380 (11)	0.87385 (10)	0.0184 (2)	
C10	0.86275 (12)	0.64296 (11)	0.98728 (11)	0.0228 (3)	
H10A	0.9199	0.7088	1.0165	0.027*	
C11	0.78122 (12)	0.60456 (12)	1.05687 (11)	0.0240 (3)	
H11A	0.7827	0.6443	1.1345	0.029*	
C12	0.69750 (12)	0.50870 (12)	1.01432 (10)	0.0214 (2)	
H12A	0.6406	0.4839	1.0616	0.026*	
C13	0.69720 (11)	0.44907 (10)	0.90242 (10)	0.0174 (2)	
H13A	0.6413	0.3823	0.8744	0.021*	

### Atomic displacement parameters (Å^2^)

**Table:**

	*U*^11^	*U*^22^	*U*^33^	*U*^12^	*U*^13^	*U*^23^
Cl	0.02888 (16)	0.03213 (19)	0.02478 (15)	−0.01301 (13)	0.01233 (12)	−0.00264 (12)
O1	0.0449 (6)	0.0315 (6)	0.0230 (4)	0.0161 (5)	0.0026 (4)	0.0071 (4)
O2	0.0329 (5)	0.0238 (5)	0.0292 (4)	0.0088 (4)	0.0161 (4)	0.0015 (4)
O3	0.0372 (5)	0.0166 (4)	0.0187 (4)	0.0056 (4)	0.0137 (3)	0.0035 (3)
N1	0.0332 (5)	0.0174 (5)	0.0147 (4)	0.0061 (4)	0.0092 (4)	−0.0015 (4)
N2	0.0217 (5)	0.0192 (5)	0.0209 (5)	0.0027 (4)	0.0045 (4)	0.0014 (4)
C1	0.0192 (5)	0.0147 (5)	0.0147 (5)	−0.0015 (4)	0.0054 (4)	−0.0022 (4)
C2	0.0259 (6)	0.0212 (6)	0.0125 (4)	0.0014 (5)	0.0066 (4)	−0.0012 (4)
C3	0.0242 (5)	0.0220 (6)	0.0129 (5)	0.0015 (5)	0.0035 (4)	0.0013 (4)
C4	0.0180 (5)	0.0151 (6)	0.0172 (5)	0.0010 (4)	0.0047 (4)	−0.0006 (4)
C5	0.0179 (5)	0.0151 (6)	0.0149 (5)	−0.0023 (4)	0.0063 (4)	−0.0030 (4)
C6	0.0182 (5)	0.0139 (5)	0.0125 (4)	−0.0011 (4)	0.0052 (4)	−0.0006 (4)
C7	0.0194 (5)	0.0150 (5)	0.0148 (5)	−0.0009 (4)	0.0076 (4)	0.0005 (4)
C8	0.0201 (5)	0.0147 (5)	0.0115 (4)	0.0026 (4)	0.0051 (4)	0.0019 (4)
C9	0.0200 (5)	0.0193 (6)	0.0165 (5)	−0.0007 (4)	0.0066 (4)	0.0009 (4)
C10	0.0257 (6)	0.0220 (6)	0.0191 (5)	−0.0029 (5)	0.0047 (4)	−0.0050 (5)
C11	0.0290 (6)	0.0271 (7)	0.0155 (5)	0.0027 (5)	0.0067 (4)	−0.0047 (5)
C12	0.0247 (6)	0.0267 (7)	0.0149 (5)	0.0023 (5)	0.0091 (4)	0.0018 (4)
C13	0.0211 (5)	0.0163 (6)	0.0150 (5)	−0.0004 (4)	0.0060 (4)	0.0016 (4)

### Geometric parameters (Å, °)

**Table:**

Cl—C9	1.7426 (12)	C5—C6	1.3972 (15)
O1—N2	1.2309 (13)	C5—H5A	0.9500
O2—N2	1.2326 (13)	C6—C7	1.4663 (15)
O3—C7	1.2322 (14)	C7—C8	1.5059 (15)
N1—C1	1.3385 (15)	C8—C9	1.3873 (16)
N1—H1A	0.8800	C8—C13	1.3975 (15)
N1—H1B	0.8800	C9—C10	1.3927 (16)
N2—C4	1.4498 (15)	C10—C11	1.3856 (18)
C1—C2	1.4201 (15)	C10—H10A	0.9500
C1—C6	1.4329 (14)	C11—C12	1.3868 (18)
C2—C3	1.3614 (17)	C11—H11A	0.9500
C2—H2A	0.9500	C12—C13	1.3882 (15)
C3—C4	1.4052 (15)	C12—H12A	0.9500
C3—H3A	0.9500	C13—H13A	0.9500
C4—C5	1.3754 (15)		
			
C1—N1—H1A	120.0	C1—C6—C7	121.40 (10)
C1—N1—H1B	120.0	O3—C7—C6	123.11 (10)
H1A—N1—H1B	120.0	O3—C7—C8	118.06 (9)
O1—N2—O2	123.10 (11)	C6—C7—C8	118.83 (10)
O1—N2—C4	118.38 (10)	C9—C8—C13	118.76 (10)
O2—N2—C4	118.52 (9)	C9—C8—C7	122.77 (9)
N1—C1—C2	119.35 (10)	C13—C8—C7	118.36 (10)
N1—C1—C6	122.63 (10)	C8—C9—C10	121.23 (10)
C2—C1—C6	118.00 (10)	C8—C9—Cl	120.08 (8)
C3—C2—C1	121.87 (10)	C10—C9—Cl	118.67 (9)
C3—C2—H2A	119.1	C11—C10—C9	119.10 (11)
C1—C2—H2A	119.1	C11—C10—H10A	120.5
C2—C3—C4	119.06 (10)	C9—C10—H10A	120.5
C2—C3—H3A	120.5	C10—C11—C12	120.62 (11)
C4—C3—H3A	120.5	C10—C11—H11A	119.7
C5—C4—C3	121.35 (11)	C12—C11—H11A	119.7
C5—C4—N2	119.28 (10)	C11—C12—C13	119.78 (11)
C3—C4—N2	119.37 (10)	C11—C12—H12A	120.1
C4—C5—C6	120.43 (10)	C13—C12—H12A	120.1
C4—C5—H5A	119.8	C12—C13—C8	120.49 (11)
C6—C5—H5A	119.8	C12—C13—H13A	119.8
C5—C6—C1	119.20 (9)	C8—C13—H13A	119.8
C5—C6—C7	119.40 (9)		
			
N1—C1—C2—C3	178.41 (11)	C1—C6—C7—O3	−14.58 (17)
C6—C1—C2—C3	−2.95 (17)	C5—C6—C7—C8	−14.71 (16)
C1—C2—C3—C4	0.38 (18)	C1—C6—C7—C8	166.26 (10)
C2—C3—C4—C5	1.95 (18)	O3—C7—C8—C9	112.56 (13)
C2—C3—C4—N2	−177.50 (11)	C6—C7—C8—C9	−68.24 (14)
O1—N2—C4—C5	179.35 (11)	O3—C7—C8—C13	−63.73 (14)
O2—N2—C4—C5	−1.01 (16)	C6—C7—C8—C13	115.48 (12)
O1—N2—C4—C3	−1.19 (17)	C13—C8—C9—C10	0.79 (17)
O2—N2—C4—C3	178.46 (11)	C7—C8—C9—C10	−175.49 (11)
C3—C4—C5—C6	−1.56 (17)	C13—C8—C9—Cl	179.06 (8)
N2—C4—C5—C6	177.89 (10)	C7—C8—C9—Cl	2.79 (15)
C4—C5—C6—C1	−1.10 (16)	C8—C9—C10—C11	−0.93 (18)
C4—C5—C6—C7	179.85 (10)	Cl—C9—C10—C11	−179.23 (10)
N1—C1—C6—C5	−178.14 (11)	C9—C10—C11—C12	−0.14 (19)
C2—C1—C6—C5	3.28 (16)	C10—C11—C12—C13	1.31 (19)
N1—C1—C6—C7	0.89 (17)	C11—C12—C13—C8	−1.45 (18)
C2—C1—C6—C7	−177.69 (10)	C9—C8—C13—C12	0.41 (17)
C5—C6—C7—O3	164.45 (11)	C7—C8—C13—C12	176.85 (10)

### Hydrogen-bond geometry (Å, °)

**Table:**

*D*—H···*A*	*D*—H	H···*A*	*D*···*A*	*D*—H···*A*
N1—H1A···O3^i^	0.88	2.22	3.0733 (12)	164
N1—H1B···O3	0.88	2.07	2.7176 (12)	130
N1—H1B···Cl^ii^	0.88	2.71	3.4848 (10)	148
C13—H13A···O2^iii^	0.95	2.46	3.1862 (14)	133

Symmetry codes: (i) *x*, −*y*+1/2, *z*−1/2; (ii) −*x*+2, *y*−1/2, −*z*+3/2; (iii) −*x*+1, *y*−1/2, −*z*+3/2.

## References

[bb1] Allen, F. H. (2002). *Acta Cryst.* B**58**, 380–388. 10.1107/s0108768102003890 12037359

[bb2] Colpaert, F. C., Wu, W. P., Hao, J. X., Royer, I., Sautel, F., Wiesenfeld-Hallin, Z. & Xu, X. J. (2004). *Eur. J. Pharmacol.* **497**, 29–33. 10.1016/j.ejphar.2004.06.026 15321732

[bb3] Cox, P. J., Anisuzzaman, A. T. Md., Skellern, G. G., Pryce-Jones, R. H., Florence, A. J. & Shankland, N. (1997). *Acta Cryst.* C**53**, 476–477.

[bb4] Cox, P. J., Kechagias, D. & Kelly, O. (2008). *Acta Cryst.* B**64**, 206–216. 10.1107/S0108768108000232 18369292

[bb5] Deleu, H., Maes, A. & Roelandts, R. (1992). *Photodermatol. Photoimmunol. Photomed.* **9**, 29–34. 1390120

[bb6] Duncan, M., Kendall, D. A. & Ralevic, V. (2004). *J. Pharmacol. Exp. Ther.* **311**, 411–419. 10.1124/jpet.104.067587 15205450

[bb7] Evans, D., Cracknell, M. E., Saunders, J. C., Smith, C. E., Willamson, W. R. N., Dowson, W. & Sweatman, W. J. F. (1987). *J. Med. Chem.* **30**, 1321–1327. 10.1021/jm00391a010 3612683

[bb8] Harrison, W. T. A., Anilkumar, H. G., Yathirajan, H. S., Sadashivamurthy, B. & Basavaraju, Y. B. (2005). *Acta Cryst.* E**61**, o4146–o4148.

[bb9] Malathy Sony, S. M., Charles, P., Ponnuswamy, M. N. & Nethaji, M. (2005). *Acta Cryst.* E**61**, o632–o634.

[bb10] Ottosen, E. R., Sorensen, M. D., Bjorkling, F., Skak-Nielsen, T., Fjording, M. S., Aaes, H. & Binderup, L. (2003). *J. Med. Chem.* **46**, 5651–5662. 10.1021/jm030851s 14667219

[bb11] Oxford Diffraction (2007). *CrysAlisPro* and *CrysAlis RED* Oxford Diffraction Ltd, Abingdon, England.

[bb12] Prasanna, M. D. & Guru Row, T. N. (2000). *CrystEngComm*, **2**, 134–140.

[bb13] Revesz, L., Blum, E., Di Padova, F. E., Buhl, T., Feifel, R., Gram, H., Hiestand, P., Manning, U. & Rucklin, G. (2004). *Bioorg. Med. Chem. Lett.* **14**, 3601–3605. 10.1016/j.bmcl.2004.03.111 15177483

[bb14] Schmidt, J. R. & Polik, W. F. (2007). *WebMO Pro* WebMO, LLC: Holland, MI, USA. URL: http://www.webmo.net.

[bb15] Sheldrick, G. M. (2008). *Acta Cryst.* A**64**, 112–122. 10.1107/S0108767307043930 18156677

[bb16] Sieroń, L., Shashikanth, S., Yathirajan, H. S., Venu, T. D., Nagaraj, B., Nagaraja, P. & Khanum, S. A. (2004). *Acta Cryst.* E**60**, o1889–o1891.

[bb17] Wiesner, J., Kettler, K., Jomaa, H. & Schlitzer, M. (2002). *Bioorg. Med. Chem. Lett.* **12**, 543–545. 10.1016/s0960-894x(01)00798-3 11844668

[bb18] Xing, Z.-Y., Liu, H.-M., Wu, L. & Zhang, W.-Q. (2005). *Acta Cryst.* E**61**, o3796–o3797.

